# Automatic diagnosis of type 2 diabetes mellitus with mild cognitive impairment using artificial intelligence based on routine T1-weighted MRI

**DOI:** 10.3389/fneur.2025.1599793

**Published:** 2025-10-09

**Authors:** Chang Li, Jun Zhang, Bo Xue, Yuwei Xia, Feng Shi, Xingyan Le, Junbang Feng, Peng Chen, Chuanming Li

**Affiliations:** ^1^Department of Medical Imaging, Chongqing Emergency Medical Center, Chongqing University Central Hospital, School of Medicine, Chongqing University, Chongqing, China; ^2^Department of Neurosurgery, Xinhua Hospital Affiliated to Shanghai Jiao Tong University School of Medicine, Shanghai, China; ^3^Shanghai United Imaging Intelligence, Shanghai, China; ^4^Department of Neurosurgery, Chongqing Key Laboratory of Emergency Medicine, Chongqing Emergency Medical Center, Chongqing University Central Hospital, Chongqing, China

**Keywords:** artificial intelligence - AI, type 2 diabetes mellitus, mild cognitive impairment - MCI, random forest model (RF), MRI

## Abstract

**Background:**

Patients with type 2 diabetes mellitus (T2DM) exhibit a heightened susceptibility to developing dementia, especially those who already present with mild cognitive impairment (MCI). Nevertheless, the fundamental etiology remains elusive, underscoring the pressing need for an objective and precise diagnostic approach in clinical settings. This study investigates the utilization of machine learning algorithms in conjunction with high-resolution sagittal T1-weighted structural imaging to facilitate automated diagnosis of T2DM patients with MCI, differentiating them from both T2DM patients without MCI and healthy controls (HCs).

**Methods:**

Thirty patients with T2DM and MCI, thirty T2DM patients without MCI, and thirty matched healthy controls (HCs) were enrolled to identify independent biomarkers and develop a diagnostic model for early cognitive impairment in T2DM. Whole-brain structural features-including cortical surface area, volume, thickness, curvature index, folding index, Gaussian curvature, mean curvature, thickness standard deviation, nuclear volume, hippocampal volume, and white matter volume-were extracted from the images of brains using automated segmentation methods. The minimum redundancy maximum relevance (MRMR) method was employed to filter out irrelevant and redundant features, reducing the dimensionality of the dataset. Subsequently, the least absolute shrinkage and selection operator (LASSO) algorithm was applied for further feature selection, ensuring the retention of only the most diagnostic features. The Random Forest (RF) classifier, a powerful machine learning model within the realm of artificial intelligence, was meticulously trained utilizing a curated feature set that had undergone rigorous refinement. To ensure the robust diagnostic performance and generalizability of the established random forest model, a 5-fold cross-validation was employed, providing a dependable estimation of the model’s effectiveness.

**Results:**

The FreeSurfer software automatically segmented the cerebral imaging data into up to 70 regions. For model establishment, a comprehensive set of 705 structural features, a series of neuropsychological tests, and standard laboratory tests were utilized. Ultimately, 8 features were selected through two feature selection strategies aimed at refining the optimal features. These included bilateral brainstem volume, left hippocampus volume, left transverse temporal gyrus volume, bilateral posterior corpus callosum volume, left medial orbitofrontal cortex Gaussian curvature, glycosylated hemoglobin, blood sugar levels, and the Digit Span Test (DST) backward score. The Random Forest (RF) model, based on the combined features, exhibited the excellent performance, with mean AUCs of 0.959 (95% CI, 0.940–0.997, mean specificity = 94.2%, mean sensitivity = 88.3%, mean accuracy = 88.3% and mean precision = 88.3%) for the training dataset and mean AUCs of 0.887 (95% CI, 0.746–0.992, mean specificity = 85.0%, mean sensitivity = 70.0%, mean accuracy = 70.0% and mean precision = 69.6%) for the testing dataset, based on 5-fold cross-validation.

**Conclusion:**

The RF model, leveraging a combination of features, demonstrates high accuracy in diagnosing T2DM with MCI. The exclusion of T2DM patients with complications may limit generalizability to the broader T2DM population, potentially inflating performance estimates. Among these features, 8 optimal indicators comprising 5 structural features, 1 neuropsychological test feature, and 2 standard laboratory test features emerge as the potential independent biomarkers for detecting early-stage cognitive impairment in T2DM patients. These features hold significant importance in understanding the pathophysiological mechanisms of T2DM-related cognitive impairment. Our fully automated model is capable of swiftly processing MRI data, enabling precise and objective differentiation of T2DM with MCI. This model significantly enhances diagnostic efficiency and holds considerable value in clinical practice, facilitating early diagnosis of T2DM with MCI.

## Introduction

Diabetes mellitus (DM) represents a chronic metabolic disorder primarily distinguished by persistently elevated blood glucose levels. It is further classified as a systemic condition capable of impacting nearly every organ system within the human body (1–3). Notable complications associated with DM encompass stroke, cardiovascular disease, peripheral arterial disease, neuropathy, retinopathy, nephropathy, and immunocompromise (4, 5). Additionally, DM can adversely affect brain tissue and cerebrovascular structures, leading to a spectrum of structural and functional disturbances within the nervous system (6, 7). In recent decades, DM has transitioned from being predominantly viewed as a metabolic ailment to a multifaceted disease entity, underscoring the increasing burden and risks posed by its emerging complications (8).

Diabetes mellitus has long been recognized as a potential precursor to widespread cognitive dysfunction (9, 10). In the specific context of type 2 diabetes mellitus (T2DM), cognitive decline manifests through a sequential progression encompassing diabetes-related cognitive decline, mild cognitive impairment (MCI), and eventually dementia (10–13). This cognitive dysfunction, characterized by impairments in memory, attention, language, and executive function, presents a formidable challenge for patients, their families, and the wider healthcare community (14). Hence, early identification of alterations in T2DM patients with Mild Cognitive Impairment (MCI) is crucial not only for optimizing patient care but also for advancing future therapeutic strategies.

The effects of T2DM on brain function and cognitive dysfunction have garnered considerable attention within the academic community. The intricate relationship between T2DM and cognitive decline is multifaceted, and although the precise mechanisms underlying this connection remain partially elusive, several potential pathological processes have come to light. Studies have highlighted hyperglycemia, vascular factors, oxidative stress, inflammation, and insulin resistance as crucial contributors to cognitive impairment in individuals with T2DM (3, 15). Furthermore, extensive research has been conducted to identify risk factors for cognitive dysfunction in T2DM patients. Numerous studies have pinpointed age, education level, hemoglobin A1c (HbA1c) levels, creatine kinase levels, severe hypoglycemia events, and malnutrition as independent predictors of cognitive impairment in T2DM patients (12, 16, 17). Additional studies have further contributed to our understanding of this complex issue by developing and validating risk scores for predicting mild cognitive impairment in T2DM patients (18, 19). The functional alterations in the brain are intrinsically tied to the structural modifications occurring across various brain regions, and these structural changes, in turn, elicit corresponding functional shifts (3, 6, 14, 20). Emerging evidence suggests that subtle neurostructural alterations may precede clinical manifestations of cognitive impairment in individuals with Type 2 Diabetes Mellitus (T2DM). Advanced neuroimaging studies have revealed microstructural brain abnormalities including disruptions in white matter integrity, gray matter atrophy, and altered functional connectivity in T2DM patients prior to the onset of overt cognitive deficits. These preclinical neuroimaging findings underscore the potential utility of biomarkers derived from magnetic resonance imaging (MRI) and related modalities for identifying individuals at elevated risk of diabetic-related cognitive decline. Notably, such biomarkers may offer critical diagnostic insights in clinical scenarios where early neurocognitive symptoms remain subclinical, underscoring their value in informing timely intervention strategies to mitigate progressive neuronal damage (21). Neuroimaging studies have demonstrated that individuals with T2DM exhibit significantly greater cerebral atrophy and vascular pathology relative to age-matched healthy controls. Of particular significance, quantitative MRI analyses reveal accelerated gray matter volume loss, cortical thinning in frontal and temporal regions critical for cognitive function, and increased amyloid-beta deposition—pathological hallmarks collectively implicated in the neurodegenerative cascade underlying diabetic-related cognitive impairment. These imaging metrics not only correlate with metabolic dysregulation, such as chronic hyperglycemia and insulin resistance, but also provide mechanistic insights into the synergistic effects of vascular damage and neurodegenerative processes on neurocognitive trajectories in T2DM populations (22–25). Furthermore, advanced neuroimaging analyses have elucidated that T2DM is associated with both generalized cortical atrophy and localized neurodegenerative alterations within specific morphometric brain networks. Of particular significance, these studies have identified overlapping neuroanatomical substrates in cognitively salient regions, most prominently within the limbic-paralimbic circuitry—a network critical for memory consolidation, emotional regulation, and cognitive control. Notably, the hippocampus, a medial temporal lobe structure integral to episodic memory encoding, and the cingulate gyrus, a key node in the salience network involved in attentional modulation, emerge as particularly vulnerable to T2DM-related neuropathology. These regional vulnerabilities are hypothesized to arise from synergistic metabolic stressors, including chronic hyperglycemia, vascular inflammation, and neuroinflammatory cascades, which collectively disrupt synaptic integrity and promote neuronal apoptosis in susceptible circuits (17, 26).

Preclinical investigations utilizing well-established rodent models, particularly diabetic rats, have provided critical insights into the neuropathological sequelae of diabetes mellitus (DM). These studies demonstrate that chronic hyperglycemia induces progressive brain atrophy, accompanied by demyelination processes characterized by myelin degradation and vacuolar disintegration of white matter tracts. Notably, such histopathological hallmarks are recapitulated across species, underscoring their translational relevance to human T2DM-related neurocognitive decline (27). These findings collectively underscore the intricate interplay between structural and functional brain changes in T2DM and their potential implications for cognitive health.

While prior investigations have dissected individual risk factors for neurocognitive dysfunction in type 2 diabetes mellitus (T2DM), critical knowledge gaps persist regarding the hierarchical importance of these variables and their interdependent relationships. Specifically, conventional analytical approaches have not systematically integrated multidimensional risk profiles, nor have they leveraged artificial intelligence (AI) methodologies to unravel the complexity of this condition. These limitations underscore the need for innovative, data-driven frameworks that can dissect the pathogenetic underpinnings of T2DM-related neurocognitive dysfunction.

To address this critical gap, our study adopts a novel AI-driven approach that harmonizes three interconnected layers of data. In the study, a comprehensive set of 705 structural features, a series of neuropsychological tests, and standard laboratory tests were utilized. By integrating these multidimensional risk factors into a diagnostic framework, an artificial intelligence (AI)-driven automatic diagnostic model is developed to enable high-fidelity, data-driven assessment of neurocognitive dysfunction in type 2 diabetes. Central to this framework is the implementation of a Random Forest (RF) classifier, an ensemble learning algorithm that overcomes the limitations of conventional models through dual innovation. First, its inherent high-dimensional data processing capability addresses feature redundancy in neuroimaging analysis, reduces overfitting risks while preserving critical biomarkers through majority voting. Second, the model’s nonlinear modeling prowess captures intricate pathophysiological interactions, through recursive partitioning of feature space, each decision tree encodes nonlinear relationships, such as the threshold effects between hippocampal atrophy and HbA1c levels, while the forest ensemble aggregates these patterns to reveal higher-order synergies between structural brain changes, metabolic dysregulation, and cognitive test performance. This model transcends conventional diagnostic paradigms by synthesizing heterogeneous clinical, metabolic, and neuroimaging variables into actionable risk profiles, ensuring both sensitivity and specificity in identifying subtle cognitive declines. Furthermore, by analyzing the optimal features in our AI automatic diagnostic model, we gain critical insights to design personalized treatment strategies. Specifically, the model identifies which modifiable risks have the greatest impact on cognitive decline in diabetes. This allows clinicians to tailor interventions to each patient’s unique profile. The AI model detects subtle cognitive changes earlier than traditional methods by pinpointing risk combinations. This multimodal AI model enables clinicians to accurately and objectively assess whether patients with type 2 diabetes mellitus have concomitant mild cognitive impairment, thereby providing robust support for subsequent precise diagnosis and treatment decisions. Meanwhile, clinicians receive a flexible framework to delay cognitive decline using timely, research-backed treatments. Ultimately, this approach showcases AI’s power to translate complex biological patterns into practical solutions, bridging the gap between lab discoveries and patient treatment.

This innovative model represents a paradigm shift in diagnostic precision, leveraging multidimensional data integration to transcend conventional diagnostic limitations. By decoding the intricate interplay between metabolic dysregulation and neurocognitive deficits, it not only accelerates the identification of preclinical diabetes-related cognitive impairment but also equips clinicians with a dynamic risk calculus framework. Such granular diagnostic stratification enables the formulation of individualized care pathways, merging pharmacotherapeutics, lifestyle optimization, and neurocognitive rehabilitation into a cohesive strategy. The model’s capacity to operationalize complex biological insights into actionable clinical intelligence heralds a new era in preventive neuroendocrinology, where timely intervention can potentially disrupt the pathogenic cascade linking hyperglycemia to dementia.

## Materials and methods

### Participants

A total of 90 participants were enrolled in this study, comprising 30 healthy controls, 30 patients with type 2 diabetes mellitus (T2DM) without mild cognitive impairment (T2DM group), and 30 patients with T2DM accompanied by mild cognitive impairment (T2DM-MCI group). Demographic and clinical data, including gender, age, and years of education, were systematically collected and analyzed for all participants. The study population was recruited from our hospital between October 2015 and June 2024, encompassing individuals with T2DM, both with and without MCI. The diagnosis of T2DM was established in accordance with the diagnostic criteria outlined by the World Health Organization (WHO). For the diagnosis of MCI, the criteria proposed by the European Alzheimer’s Disease Consortium were applied. This criteria included the following parameters: Mini-Mental State Examination (MMSE) score > 24, Clinical Dementia Rating (CDR) score ≥ 0.5, Montreal Cognitive Assessment (MoCA) score < 26, normal activities of daily living (ADL) scores, and subjective complaints of memory impairment. All participants underwent structural MRI scanning as well as comprehensive neurological and neuropsychological evaluations. Prior to the commencement of the study, written informed consent was obtained from each participant. Only right-handed individuals were included. Participants with a history of brain injury, major depression, Parkinson’s disease, alcoholism, epilepsy, or any other psychiatric or neurological disorder were excluded from the study. Additionally, individuals with contraindications to MRI, severe claustrophobia, dementia (Mini-Mental State Examination score ≤ 24), or severe depression (Hamilton Depression Rating Scale score ≥ 18) were also excluded. In our study, patients with T2DM who exhibited microvascular complications, specifically neuropathy, retinopathy, and nephropathy were excluded from participation. We enrolled thirty volunteers as healthy controls (HC), who reported no vascular risk factors, nervous system diseases, psychiatric illnesses, or cognitive complaints. For each individual, weight, height, and body mass index (BMI) were meticulously measured. This study was approved by the Medical Ethics Committee of our institution and was conducted in strict adherence to the principles of the Declaration of Helsinki.

### Neuropsychological assessments

The neuropsychological assessments encompassed a comprehensive battery of tests, including the Mini-Mental State Examination (MMSE), Montreal Cognitive Assessment (MoCA), Hamilton Depression Scale (HAMD), Digit Symbol Coding Test (DSCT), Digit Span Test (DST), Verbal Fluency Test (VFT), Rey-Osterrieth Complex Figure Test (ROCF), Auditory Verbal Learning Test (AVLT), Trail-Making Test (TMT).

### Standard laboratory tests

Standard laboratory analyses were conducted to assess a comprehensive panel of biomarkers, including glycosylated hemoglobin (HbA1c), fasting C-peptide, fasting insulin, fasting plasma glucose (FPG), triglycerides (TG), total cholesterol (TC), low-density lipoprotein (LDL), high-density lipoprotein (HDL), blood urea nitrogen (BUN), homocysteine, urinary microalbumin, uric acid, cystatin C, serum creatinine, thyroid stimulating hormone (TSH), free triiodothyronine (FT3), and free thyroxine (FT4).

### MR image acquisition

All magnetic resonance imaging (MRI) examinations were performed using a 3-Tesla Trio MRI scanner (Siemens Healthcare, Erlangen, Germany) with a 12-channel phased-array head coil. Participants were instructed to maintain a supine position, remain motionless, and keep their eyes closed throughout the imaging procedure. The three-dimensional High-resolution structural images were obtained using a T1-weighted magnetization-prepared rapid acquisition gradient echo (MPRAGE) sequence with the following parameters: inversion time (TI) = 900 ms, echo time (TE) = 2.52 ms, repetition time (TR) = 1,900 ms, flip angle = 9°, 176 slices, slice thickness = 1.0 mm, matrix size = 256 × 256, and voxel size = 1 × 1 × 1 mm^3^. Subsequently, conventional brain T1-weighted imaging (TE/TR = 2.78/200 ms, flip angle = 70°, 25 slices, slice thickness = 4.0 mm, matrix size = 384 × 384, voxel size = 0.7 × 0.6 × 5 mm^3^) and fluid-attenuated inversion recovery (FLAIR) imaging (TI/TE/TR = 2500/93/9000 ms, flip angle = 130°, 25 slices, slice thickness = 4.0 mm, matrix size = 256 × 256, voxel size = 0.9 × 0.9 × 4 mm^3^) were performed on all subjects to exclude potential white matter abnormalities and organic brain lesions.

### Image processing

The MRI scanner-acquired data were exported to a dedicated image-processing workstation, and subsequent offline analysis was conducted utilizing the Linux Operating System. Prior to any further examination of the 3D brain images, all images were meticulously verified to ensure they were unaffected by head motion. Subsequently, the data were converted into the MGZ format (a compressed file format developed by Massachusetts General Hospital). Utilizing FreeSurfer software (version 5.3.0, accessible at http://surfer.nmr.mgh.harvard.edu), we measured the structural features encompassing cortical surface area, volume, thickness, curvature index, folding index, Gaussian curvature, mean curvature, thickness standard deviation, nuclear volume, hippocampal volume, and white matter volume. The automated processing stream of FreeSurfer encompassed several crucial steps, including spatial normalization via Talairach coordinate transformation, skull stripping using hybrid watershed/surface deformation algorithm, topological defect correction with spherical inflation-based surface reconstruction, inflation of the folded surface, and registration into an average spherical surface template. To achieve sub-millimeter precision in segmenting gray/white matter tissue and cerebrospinal fluid (CSF), a deformable surface algorithm was employed.

### Features selection

A robust two-step feature selection methodology was implemented, integrating Minimum Redundancy Maximum Relevance (MRMR) and Least Absolute Shrinkage and Selection Operator (LASSO) to identify the most diagnostic features. The process was incorporated into a 5-fold cross-validation framework, where the data were divided into training and testing sets. Features selected based on the training sets were aggregated by voting to determine the final set used in the model. Initially, MRMR was applied to the training sets in each fold of the 5-fold cross-validation, selecting the top 10 features most relevant to the target variable while minimizing redundancy. Subsequently, LASSO was applied to these features for further refinement within each fold, using an L1 regularization term to promote sparsity by shrinking some coefficients to zero. This comprehensive approach ensured the retention of only the most diagnostic features, thus enhancing model performance and interpretability. The final set of features was determined based on the voting results from all training folds, identifying the most consistently selected features across them.

### Models building and evaluation

Following the feature selection, the Random Forest (RF) classifier, a powerful machine learning model within the realm of artificial intelligence, was meticulously trained utilizing a curated feature set that had undergone rigorous refinement. The model architecture was configured with 100 decision trees (ntree = 100) to ensure convergence of error estimation, with maximum tree depth constrained to 2 levels (max_depth = 2) to prevent overfitting. Notably, a 5-fold cross-validation approach was implemented, offering a robust estimation of model utility. Following this cross-validation, the average accuracy and Receiver Operating Characteristic (ROC) curves were computed. This method provided a robust and unbiased assessment of the model’s diagnostic capabilities, thereby ensuring a comprehensive and objective evaluation of its utility.

### Statistical analyses

Statistical analyses were conducted using IBM SPSS Statistics (version 20.0; Armonk, NY). The Kolmogorov–Smirnov test was employed to assess data distribution normality. For normally distributed variables, intergroup comparisons were performed using one-way analysis of variance (ANOVA), with *post hoc* Bonferroni-corrected comparisons conducted following significant ANOVA results. Non-normally distributed variables were analyzed using the Kruskal-Wallis test. Categorical data were evaluated using Chi-square tests. Both feature selection and machine learning model construction were established on the uAI Research Portal (28). Subsequently, the diagnostic value of each model was assessed through the Areas Under the Curves (AUCs). Specifically, an AUC exceeding 0.9 was indicative of excellent diagnostic efficacy; an AUC ranging from 0.7 to 0.9 suggested good diagnostic efficacy; an AUC between 0.5 and 0.7 implied poor diagnostic efficacy; and an AUC not surpassing 0.5 signified the absence of diagnostic value.

## Results

Demographic characteristics, clinical parameters, and neuropsychological data of the T2DM-MCI, T2DM, and HC cohorts are summarized in Tables 1, 2. The three groups exhibited comparable baseline profiles, with no statistically significant differences observed in age, sex distribution, educational attainment, systolic/diastolic blood pressure. The entire brains were analyzed using automated segmentation methods to extract an extensive dataset comprising 705 structural features. These features encompassed a variety of metrics, including cortical surface area, volume, thickness, curvature index, folding index, Gaussian curvature, mean curvature, thickness standard deviation, nuclear volume, hippocampal volume, and white matter volume. To facilitate model establishment, a comprehensive dataset was organized, incorporating this extensive set of structural features, along with a series of neuropsychological data and standard laboratory data.

**Table 1:** Demographic and clinical characteristics of T2DM-MCI, T2DM, and HC groups.

Data	T2DM-MCI (*n* = 30)	T2DM (*n* = 30)	HC (*n* = 30)	*F*-value (*t*/χ^2^)	*p* value
Demographic data					
Age (years)	55.9 ± 6.54	54.97 ± 5.54	53.17 ± 6.57	1.491	0.231
Gender (male/female)	12/18	19/11	14/16	3.467	0.177^a^
Education (years)	10.43 ± 2.94	11.90 ± 2.92	11.80 ± 2.99	2.316	0.105
Diabetes duration (years)	6.93 ± 5.46	7.93 ± 5.98	—	0.699	0.933
Clinical data					
HbA1c (%)	9.13 ± 2.07	8.84 ± 1.72	5.50 ± 0.36	49.505	<0.001* ^#^
Fasting glucose (mmol/L)	9.14 ± 3.05	8.56 ± 1.97	5.47 ± 0.63	25.769	<0.001* ^#^
BMI (kg/m^2^)	25.59 ± 3.31	24.08 ± 3.02	23.45 ± 2.59	4.052	0.021*
LDL cholesterin (mmol/L)	3.36 ± 1.06	2.93 ± 0.78	3.10 ± 0.67	1.874	0.160
HDL cholesterin (mmol/L)	1.16 ± 0.33	1.18 ± 0.30	1.40 ± 0.33	5.167	0.008* ^#^
Total cholesterin (mmol/L)	5.24 ± 1.45	4.96 ± 1.37	5.21 ± 0.96	0.432	0.651
Diastolic blood pressure (mmHg)	80.60 ± 10.33	82.90 ± 9.80	79.77 ± 9.17	0.895	0.441
Systolic blood pressure (mmHg)	130.57 ± 18.25	131.37 ± 14.78	128.13 ± 18.10	0.194	0.749

All subjects (T2DM-MCI, T2DM, HC) were matched for age, gender, and education. Values are the mean ± standard deviation. ^a^Chi-square test for gender. The comparisons of demographic and clinical data among three groups were performed with ANOVA. The level of significance for intergroup differences was set at *p* < 0.05. *Denotes *p* < 0.05 T2DM-MCI vs. HC with *post hoc* test, Bonferroni corrected. ^#^Denotes *p* < 0.05 T2DM vs. HC with *post hoc* test, Bonferroni corrected. No significant difference was shown between T2DM and T2DM-MCI with *post hoc* test, Bonferroni corrected. BMI, body mass index; HbA1c, glycosylated hemoglobin; HDL, high-density lipoprotein; LDL, low-density lipoprotein.

**Table 2:** Comparison of the neuropsychological test results among T2DM-MCI, T2DM and HC groups.

Test	T2DM-MCI (*n* = 30)	T2DM (*n* = 30)	HC (*n* = 30)	*F*-value (*t*/χ^2^)	*p* value
MMSE	27.93 ± 1.31	28.43 ± 1.07	28.43 ± 1.17	1.774	0.176
MoCA	22.93 ± 1.95	27.00 ± 0.83	27.77 ± 1.28	99.370	<0.001* ^#^
DST-forwards	8.93 ± 1.08	8.87 ± 0.82	9.57 ± 1.38	3.584	0.032
DST-backwards	4.67 ± 0.88	5.03 ± 0.89	5.53 ± 1.17	5.805	0.004*
TMT-A	63.80 ± 21.66	51.97 ± 17.44	48.57 ± 16.61	5.484	0.006* ^#^
TMT-B	80.77 ± 26.59	67.73 ± 24.19	61.33 ± 22.74	4.877	0.010*
WAIS	35.63 ± 8.33	41.23 ± 10.83	46.23 ± 11.99	7.658	0.001*
ROCF-immediate recall	19.00 ± 6.64	22.63 ± 6.95	24.08 ± 8.49	3.754	0.027*
ROCF-copy	31.97 ± 3.88	32.55 ± 4.11	33.17 ± 1.90	0.911	0.406
ROCF-delayed recall (20 min)	18.20 ± 6.19	21.85 ± 7.13	23.65 ± 7.74	4.659	0.012*
VFT	40.60 ± 7.04	44.10 ± 8.29	44.83 ± 6.06	2.970	0.057
AVLT-immediately recall	19.50 ± 4.55	22.77 ± 3.83	22.83 ± 5.00	5.406	0.006* ^#^
AVLT-recognition	21.10 ± 3.60	21.87 ± 1.48	22.90 ± 1.45	4.252	0.017*
AVLT-delayed recall (5 min)	7.23 ± 2.40	7.80 ± 1.56	8.20 ± 1.94	1.775	0.176
AVLT-delayed recall (20 min)	6.83 ± 2.64	7.33 ± 1.77	7.87 ± 2.18	1.620	0.204

Values are the mean ± standard deviation. The comparisons of each neuropsychological test among three groups were performed with ANOVA. The level of significance for intergroup differences was set at *P* < 0.05. *Denotes *p* < 0.05 T2DM-MCI vs. HC with *post hoc* test, Bonferroni corrected. ^#^Denotes *p* < 0.05 T2DM-MCI vs. T2DM with *post hoc* test, Bonferroni corrected. No significant difference was shown between T2DM and HC with *post hoc* test, Bonferroni corrected. MMSE, mini-mental state examination; MoCA, montreal cognitive assessment; DST, digital span test; TMT, trail making test; WAIS, Wechsler adult intelligence scale; ROCF, Rey-Osterrieth complex figure; VFT, verbal fluency test; AVLT, auditory verbal learning test.

**Figure 1:**
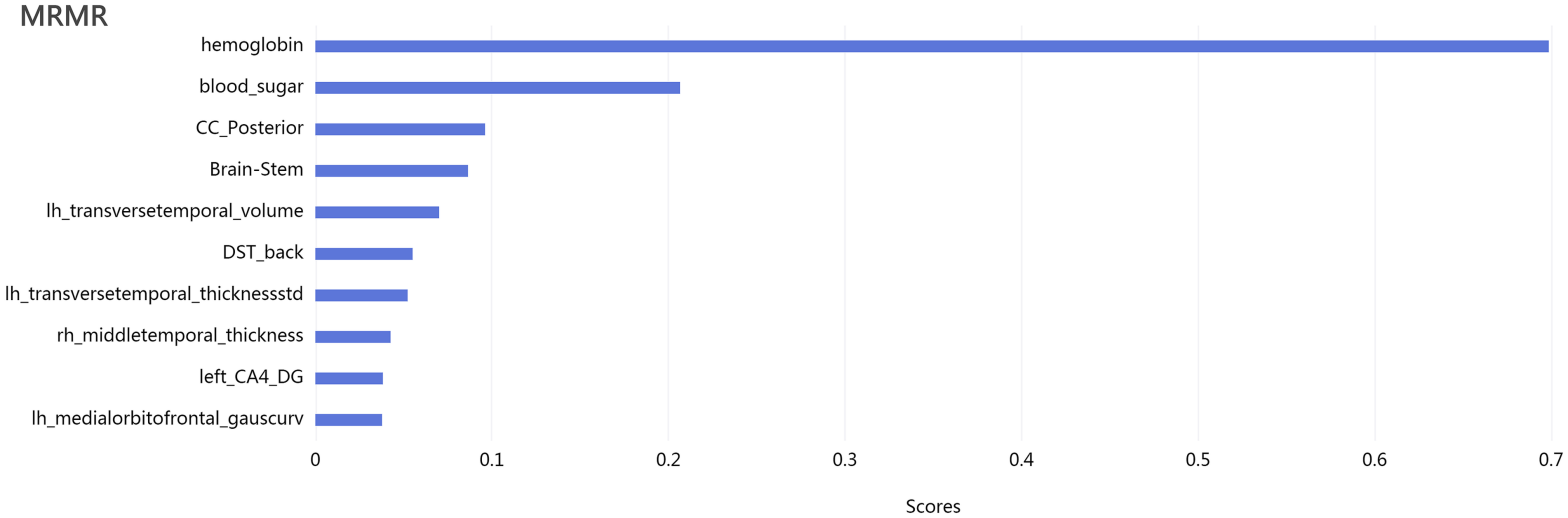
The maximum relevance-minimum redundancy (mRMR) algorithm, reducing irrelevant and redundant data.

**Figure 2:**
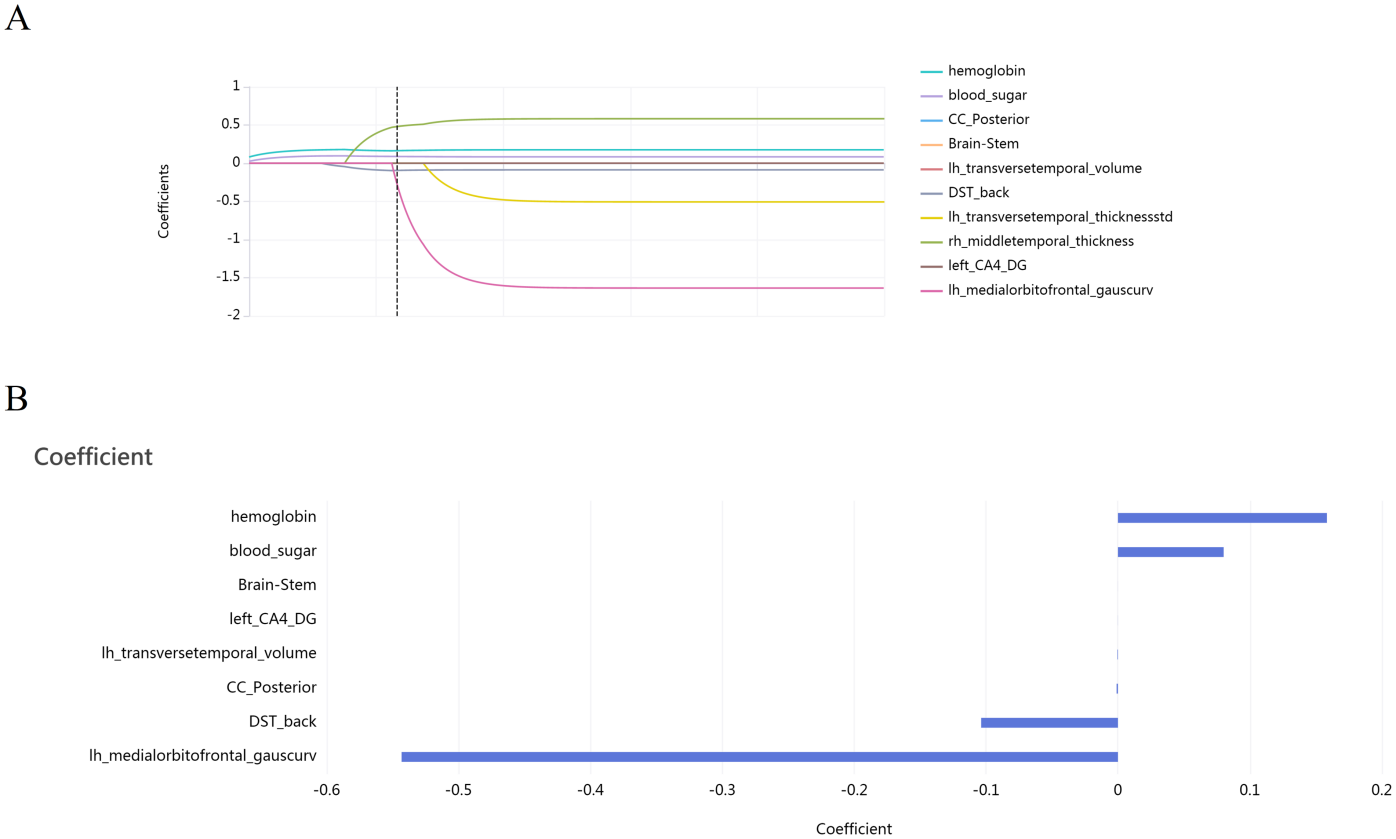
After applying the LASSO feature selection method, a total of 8 optimal features were retained (*p* < 0.05). **(A)** The least absolute shrinkage and selection operator (LASSO) algorithm, highlighting feature selection through regularization. **(B)** LASSO coefficients and corresponding optimal features.

To refine the dataset and enhance model performance, the minimum redundancy maximum relevance (MRMR) method was employed to eliminate irrelevant and redundant features (Figure 1). Subsequently, the least absolute shrinkage and selection operator (LASSO) algorithm was applied for further feature selection, ensuring that only the optimal features were retained (Figure 2).

**Figure 3:**
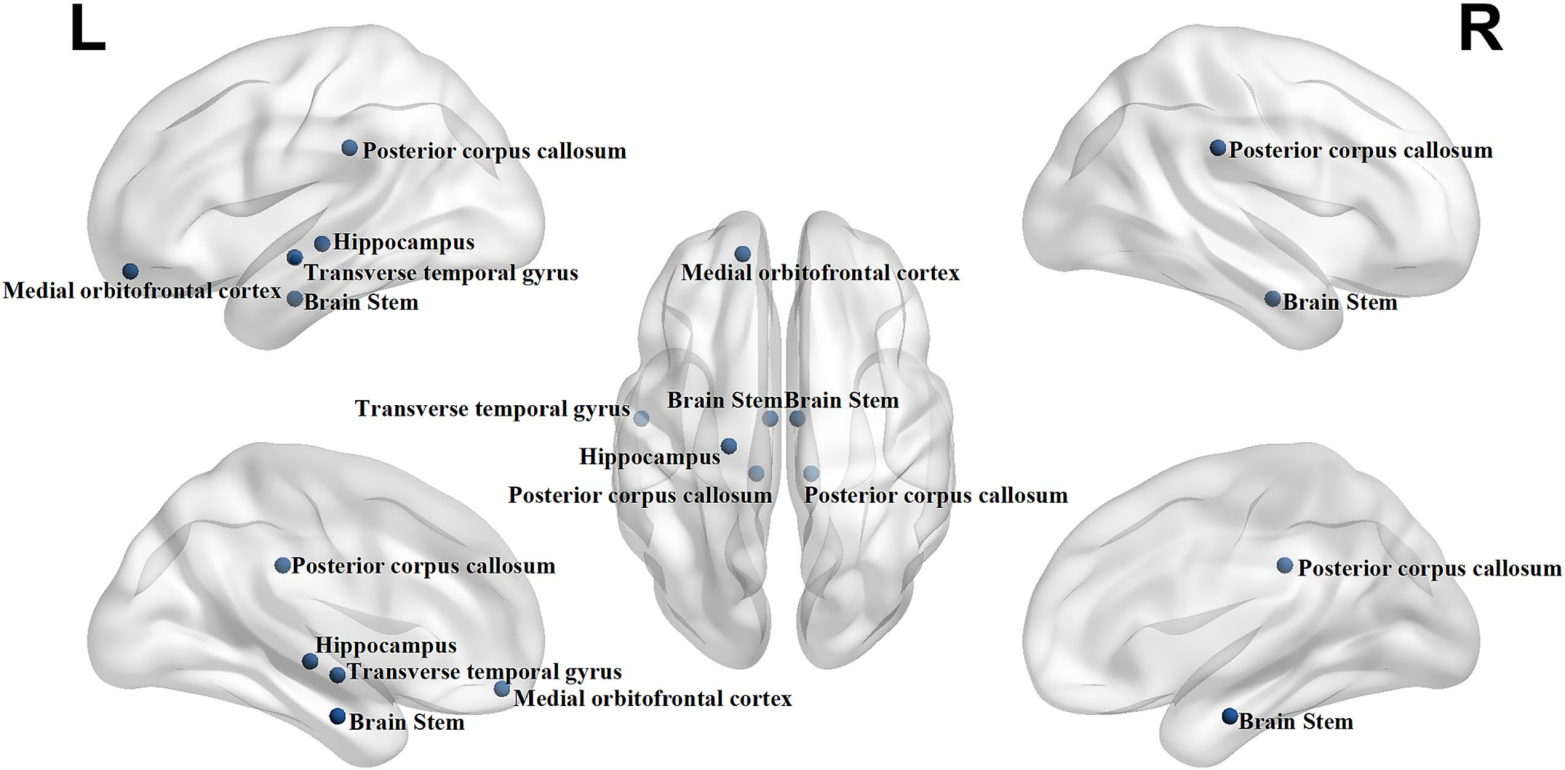
The optimal structural features were selected including bilateral brainstem volume, left hippocampus volume, left transverse temporal gyrus volume, bilateral posterior corpus callosum volume, left medial orbitofrontal cortex Gaussian curvature.

Through these two rigorous feature selection strategies, eight optimal features were ultimately selected including bilateral brainstem volume, left hippocampus volume, left transverse temporal gyrus volume, bilateral posterior corpus callosum volume, left medial orbitofrontal cortex Gaussian curvature, glycosylated hemoglobin levels, blood sugar levels, and the Digit Span Test (DST) backward score. The optimal structural features were shown in Figure 3.

**Figure 4:**
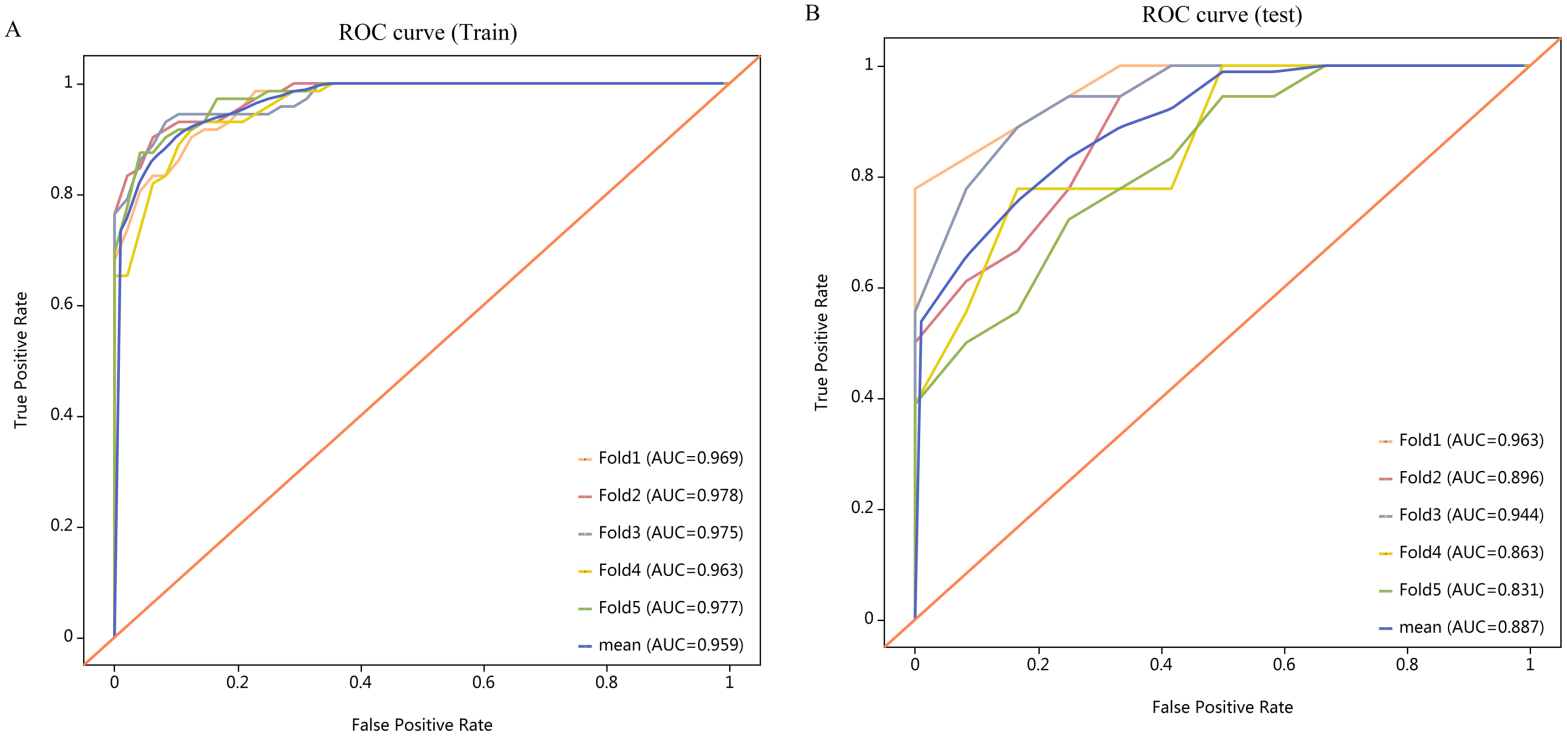
ROC curves illustrating the performance of the random forest model using combined features via 5-fold cross-validation. **(A)** The ROC curve depicting the performance of the random forest model on the training dataset. **(B)** The ROC curve depicting the performance of the random forest model on the testing dataset.

The Random Forest (RF) model, based on the combined features, exhibited the excellent performance, with mean AUCs of 0.959 (95% CI, 0.940–0.997, mean specificity = 94.2%, mean sensitivity = 88.3%, mean accuracy = 88.3% and mean precision = 88.3%) for the training dataset and mean AUCs of 0.887 (95% CI, 0.746–0.992, mean specificity = 85.0%, mean sensitivity = 70.0%, mean accuracy = 70.0% and mean precision = 69.6%) for the testing dataset, based on 5-fold cross-validation (Table 3). The receiver operating characteristic (ROC) analysis revealed superior discriminative capacity and the unwavering robustness of the random forest model (Figure 4).

**Table 3:** Diagnostic value of the random forest (RF) model based on the combined features.

Method	AUC (train)	AUC (test)	Sensitivity (train)	Sensitivity (test)	Specificity (train)	Specificity (test)	Accuracy (train)	Accuracy (test)	Precision (train)	Precision (test)	f1 Score (train)	f1 Score (test)
Random forest	0.959	0.887	0.883	0.7	0.942	0.85	0.883	0.7	0.883	0.696	0.882	0.694

AUC, areas under the curves.

## Discussion

This study demonstrates the feasibility of developing an artificial intelligence (AI) framework utilizing routine T1-weighted MRI, clinical biomarkers, and neuropsychological scales to differentiate type 2 diabetes mellitus (T2DM) patients with mild cognitive impairment (MCI), non-cognitively impaired T2DM individuals, and healthy controls. We identified eight characteristic signatures reflecting the multifactorial pathophysiology of T2DM-MCI. Notably, the combined features of bilateral brainstem volume reduction, left hippocampal atrophy, diminished left transverse temporal gyrus volume, and posterior corpus callosum thinning potentially align with established neurodegeneration patterns in metabolic disorders. The inclusion of glycosylated hemoglobin and blood glucose levels further underscores the potential interaction between chronic hyperglycemia and neural degeneration (29–31). The Random Forest (RF) model exhibited robust diagnostic performance (training AUC = 0.959, testing AUC = 0.887), emphasizing the complementary value of integrating structural MRI metrics, metabolic, and neuropsychological data. The RF model demonstrates exceptional capability in automatically capturing nonlinear relationships and higher-order interactions among variables through its hierarchical cascade structure of decision trees, eliminating the need for stringent assumptions regarding data distributions. Particularly evident in its capacity to synergistically integrate structural MRI metrics, metabolic parameters, and neuropsychological test outcomes—a critical requirement for deciphering the complex pathophysiological interplay in diabetic cognitive impairment.

The structural alterations identified in T2DM-MCI patients predominantly localize to hub regions of the default mode network (DMN) and executive control network (ECN), offering insights into the neural mechanisms of diabetes-associated cognitive decline. Reduced left hippocampal volume, a hallmark feature in our study, is a critical node of the DMN, which is responsible for episodic memory and self-referential processing (32). Hippocampal atrophy in T2DM may arise from chronic hyperglycemia-induced oxidative stress and impaired insulin signaling, disrupting synaptic plasticity and promoting amyloid-*β* deposition (33). This aligns with findings from Gold et al. (34) who demonstrated reduced hippocampal volume in diabetic patients correlating with poor glycemic control. Along with structural changes in the hippocampus, there could be also abnormalities in its functional properties. The study by Chen et al. (35) revealed a significant decrease in spontaneous brain activity in the hippocampal region of patients with cognitive impairment. In addition, the multimodal meta-analysis revealed complex conjoint and dissociated alterations in brain structure and function in patients with T2DM-MCI, primarily involving the DMN, limbic system, cerebellum, insula, and visual cortex (36).

Concurrent thinning of the bilateral posterior corpus callosum, a major white matter tract connecting parietal-occipital regions, likely impairs interhemispheric communication within the ECN and dorsal attention network (DAN), exacerbating deficits in working memory and attentional control (37). The observed reduction in left transverse temporal gyrus volume and left medial orbitofrontal cortex (mOFC) curvature anomalies implicate dysfunction in multisensory integration and salience network (SN) regulation (38). The transverse temporal gyrus is not only central to auditory processing but also contributes to higher-order cognitive functions through its connections to frontoparietal networks (39). Atrophy in this region may reflect early neurodegeneration in parieto-temporal circuits, compounded by diabetes-related microvascular damage. Meanwhile, abnormal gaussian curvature in the mOFC, a key SN node, may suggest altered cortical folding patterns due to metabolic stress. The mOFC integrates visceral and emotional stimuli, and its structural anomalies may disrupt SN-ECN interactions, impairing decision-making and error monitoring (40). Notably, the inclusion of the Digit Span Test (DST) backward score - a measure of working memory - further underscores ECN inefficiency, potentially tied to frontal-striatal circuit disruption from hyperglycemia-induced white matter lesions.

The bilateral brainstem volume reduction highlights the vulnerability of neuromodulatory nuclei to diabetic pathophysiology, particularly within monoaminergic systems. The brainstem houses serotonergic (raphe nuclei) and noradrenergic (locus coeruleus) neurons that regulate SN activity and arousal (41). Volume loss in these regions may diminish the brain’s capacity to prioritize salient stimuli, contributing to attentional deficits in T2DM-MCI. This aligns with diffusion tensor imaging studies showing diabetes-associated axonal degeneration in corticospinal tracts (42). Furthermore, elevated glycosylated hemoglobin (HbA1c) and blood glucose levels likely exacerbate neurodegeneration through advanced glycation end-product (AGE) accumulation and blood–brain barrier dysfunction, particularly in glucose-sensitive regions like the hippocampus (43). Chronic hyperglycemia accelerates cerebral hypometabolism in frontotemporal networks, mirroring Alzheimer’s-like metabolic patterns (44).

Chronic hyperglycemia and elevated HbA1c levels likely drive cognitive impairment in T2DM-MCI through multiple synergistic pathways, including AGE accumulation, oxidative stress, and blood–brain barrier (BBB) dysfunction (44, 45). Prolonged hyperglycemia accelerates the formation of AGEs, which crosslink with extracellular matrix proteins, impairing neuronal plasticity and microvascular integrity (45). AGEs also bind to their receptors (RAGE) on endothelial cells, triggering pro-inflammatory cascades that exacerbate neuroinflammation and synaptic loss, particularly in the hippocampus and prefrontal cortex - regions critical for memory and executive function (45). The studies demonstrated that AGE accumulation in T2DM patients correlates with reduced gray matter volume and cognitive decline (6, 46, 47). Concurrently, hyperglycemia-induced oxidative stress depletes endogenous antioxidants, leading to mitochondrial dysfunction and neuronal apoptosis, as observed in diabetic rodent models (48). Hyperglycemia further disrupts cerebral hemodynamics and BBB permeability, fostering a neurotoxic milieu. Elevated blood glucose levels impair endothelial function, reducing cerebral blood flow and causing chronic hypoxia in glucose-sensitive regions like the hippocampus (49). BBB leakage, mediated by tight junction protein degradation, allows influx of neurotoxic plasma components (e.g., fibrinogen, thrombin), which activate microglia and astrocytes, driving neuroinflammation (50). This process is compounded by insulin resistance, which diminishes neuroprotective insulin signaling pathways essential for neuronal survival and tau phosphorylation regulation (33). Brundel et al. (23) reported that T2DM patients exhibit accelerated white matter hyperintensities and cortical thinning, paralleling Alzheimer’s disease-like neurodegeneration. The bilateral posterior corpus callosum thinning observed in our study may thus reflect BBB breakdown and Wallerian degeneration due to sustained metabolic insult. The interplay between metabolic dysregulation and structural brain changes may propagate a vicious cycle of cognitive decline. Hyperglycemia-induced hippocampal atrophy disrupts the DMN, impairing memory consolidation, while prefrontal cortex damage undermines ECN efficiency. Reduced brainstem volume, as identified in our model, may dysregulate monoaminergic systems (e.g., serotonin, norepinephrine), impairing SN-mediated attention and error monitoring. Notably, the DST backward score - a marker of working memory - correlates with frontal-striatal circuit integrity, which is vulnerable to glucose toxicity. These network-level disruptions align with the “common soil” hypothesis, positing shared vascular and inflammatory pathways between T2DM and neurodegeneration. Future interventions targeting AGE inhibitors, antioxidant pathways, or BBB stabilization may mitigate diabetes-associated cognitive decline.

The machine learning framework developed in our study demonstrates that a strategically curated combination of neuroanatomical, metabolic, and neuropsychological features holds substantial discriminative power for early T2DM-MCI detection. The eight optimal features selected through MRMR-LASSO - spanning bilateral brainstem volume, left hippocampal atrophy, and glycosylated hemoglobin levels - reflect a multimodal pathophysiology that single-modality approaches might overlook. Notably, the integration of structural MRI metrics (e.g., left medial orbitofrontal cortex gaussian curvature) with biochemical markers (HbA1c, blood glucose) and the DST backward score aligns with emerging evidence that diabetes-related cognitive decline arises from synergistic interactions between neural degeneration and metabolic dysregulation. The parameters for the Random Forest (RF) model, including the number of decision trees (100) and maximum tree depth (2), were rigorously optimized through an automated hyperparameter tuning algorithm (51) in training dataset. By leveraging a widely recognized Python package for hyperparameter tuning, this methodology ensures reproducibility and alignment with best practices in machine learning. This data-driven approach ensured that the final model parameters were objectively optimized for both diagnostic performance and clinical applicability. The RF model’s robustness (training AUC = 0.959, testing AUC = 0.887) underscores the clinical viability of AI-driven tools for parsing complex, high-dimensional datasets. Its superior specificity (94.2% in training, 85.0% in testing) suggests particular utility in minimizing false positives during early screening, a critical advantage given the subtlety of T2DM-MCI manifestations.

Our study still had several limitations. Our study’s sample size was relatively small, attributable to stringent inclusion criteria. Though this situation bolstered cohort homogeneity, it potentially curtailed findings’ generalizability. The model’s sensitivity, specificity, and generalizability in actual clinical settings are likely to encounter challenges and limitations. The exclusion of T2DM patients with microvascular complications (e.g., neuropathy, retinopathy, nephropathy) undoubtedly introduces spectrum bias. While this exclusion was necessary in our initial proof-of-concept study to control for potential confounding effects and isolate the relationship between T2DM, brain structure, and cognitive status, the exclusion of T2DM patients with complications might limit generalizability to the broader T2DM population, potentially inflating performance estimates. Our cohort represents a ‘cleaner’ phenotypic subgroup, and consequently, the excellent diagnostic performance reported here may be over-optimistic when applied to the broader, more heterogeneous T2DM population seen in clinical practice, which includes patients with a wide range of comorbidities and complications. Therefore, it is critically important to emphasize that external validation in independent, larger, and more representative cohorts—specifically those including T2DM patients across the full spectrum of disease severity and complication profiles—is an essential next step. Such validation is a mandatory prerequisite for any future clinical translation of this model. In our future studies, we would utilize a larger sample size to further substantiate the findings of our current research. The disparity between training and testing performance highlights the need for external validation in diverse cohorts. While our current model has already demonstrated strong performance and broad applicability, we intend, in future work, to further consolidate its generalizability by incorporating additional multicentre datasets for external validation. The model’s reliance on routine T1-weighted MRI enhances scalability but may exclude dynamic functional or microstructural insights from advanced modalities like diffusion tensor imaging. Future research could integrate longitudinal HbA1c trajectories or amyloid-PET data to refine diagnostic accuracy, while also exploring the mechanisms in depth. In the follow-up study, a longitudinal study incorporating both MCI patients and the patients who revert from T2DM with MCI to T2DM alone will be conducted to investigate the diagnostic potential of our Random Forest model. Acknowledging the limitations of this study is crucial. The relatively modest sample size, although comparable to earlier pioneering studies in this specific field, inherently limits the generalizability of our findings and may introduce instability in the feature selection process. While the repeated cross-validation strategy and the reported confidence intervals help provide a more robust assessment under this constraint, the performance estimates would benefit from validation in a larger, independent cohort. Therefore, a primary direction for our future work will be to recruit a larger number of participants to further validate and refine the proposed model, enhance the stability of the identified biomarkers, and improve the overall generalizability of the results.

## Conclusion

The Random Forest model exhibits remarkable diagnostic accuracy for T2DM patients with MCI. The external validation in more representative cohorts including patients with complications is essential before clinical implementation. Within this feature set, eight optimal indicators have been identified as potential independent biomarkers for early-stage cognitive impairment detection in T2DM. These indicators consist of five structural features, one neuropsychological test feature, and two standard laboratory test features, all of which are crucial for elucidating the pathophysiological mechanisms of T2DM-related cognitive impairment. Our fully automated RF model demonstrates the capability to rapidly process magnetic resonance imaging data, thereby enabling precise and objective differentiation of T2DM patients with MCI from HCs and T2DM patients. This advancement not only significantly enhances diagnostic efficiency but also holds substantial promise for clinical application, offering a valuable tool for the early diagnosis of cognitive impairment in T2DM patients.

## Data Availability

The original contributions presented in the study are included in the article/supplementary material, further inquiries can be directed to the corresponding author/s.
